# TMPRSS2 Independency for Haemagglutinin Cleavage *In Vivo* Differentiates Influenza B Virus from Influenza A Virus

**DOI:** 10.1038/srep29430

**Published:** 2016-07-08

**Authors:** Kouji Sakai, Yasushi Ami, Noriko Nakajima, Katsuhiro Nakajima, Minori Kitazawa, Masaki Anraku, Ikuyo Takayama, Natthanan Sangsriratanakul, Miyuki Komura, Yuko Sato, Hideki Asanuma, Emi Takashita, Katsuhiro Komase, Kazuaki Takehara, Masato Tashiro, Hideki Hasegawa, Takato Odagiri, Makoto Takeda

**Affiliations:** 1 Department of Virology 3, National Institute of Infectious Diseases, Tokyo 208-0011, Japan; 2 Division of Experimental Animal Research, National Institute of Infectious Diseases, Tokyo 208-0011, Japan; 3 Department of Pathology, National Institute of Infectious Diseases, Tokyo 162-8640, Japan; 4 Laboratory of Animal Health, Department of Veterinary Medicine, Faculty of Agriculture, Tokyo University of Agriculture and Technology, Tokyo 183-8509, Japan; 5 Department of Microbiology, School of Medicine, Yokohama City University, Yokohama 236-0004, Japan; 6 Influenza Virus Research Center, National Institute of Infectious Diseases, Tokyo 208-0011, Japan

## Abstract

Influenza A and B viruses show clear differences in their host specificity and pandemic potential. Recent studies have revealed that the host protease TMPRSS2 plays an essential role for proteolytic activation of H1, H3, and H7 subtype strains of influenza A virus (IAV) *in vivo*. IAV possessing a monobasic cleavage site in the haemagglutinin (HA) protein replicates poorly in TMPRSS2 knockout mice owing to insufficient HA cleavage. In the present study, human isolates of influenza B virus (IBV) strains and a mouse-adapted IBV strain were analysed. The data showed that IBV successfully underwent HA cleavage in TMPRSS2 knockout mice, and that the mouse-adapted strain was fully pathogenic to these mice. The present data demonstrate a clear difference between IAV and IBV in their molecular mechanisms for spreading *in vivo*.

Influenza B virus (IBV) is classified into the same family, *Orthomyxoviridae*, as influenza A virus (IAV)^1^. Both viruses cause seasonal influenza, and the clinical manifestations of the infections are indistinguishable. However, they differ in their antigenic diversities and host ranges. IAV has 18 haemagglutinin (HA) and 11 neuraminidase (NA) subtypes, whereas no antigenic subtypes are found for IBV^1^,^2^,^3^. Only two antigenically distinct virus lineages, B/Victoria/2/87 [Victoria] and B/Yamagata/16/88 [Yamagata], have been co-circulating worldwide since 1983^4^,^5^. Although the natural hosts of IAV are wild aquatic birds, many subtypes of IAV are maintained in various birds and mammals, including humans^1^. IAV was also detected in bats^2^,^3^. By contrast, IBV mainly circulates in humans, and occasionally in seals^6^ and pigs^7^. To date, the molecular bases generating the biological differences between IAV and IBV are poorly understood.

The HA proteins of both IAV and IBV are synthesized as inactive precursors, and their proteolytic cleavage is a prerequisite for the HA conformational changes essential for virus infectivity. Recent studies using *TMPRSS2* gene knockout (TMPRSS2 KO) mice have revealed that IAV strains possessing a monobasic cleavage site in HA primarily undergo proteolytic activation by TMPRSS2, a type II transmembrane serine protease (TTSP), *in vivo*^8^,^9^,^10^. H3N2, H1N1, and H7N9 IAV strains replicated very poorly in TMPRSS2 KO mice owing to insufficient HA cleavage in these mice^8^,^9^,^10^. Therefore, TMPRSS2 is a potential molecular target for the development of anti-IAV drugs. Meanwhile, cell culture analyses demonstrated that TMPRSS2 also has the potential to activate IBV^11^. In this study, the role of TMPRSS2 for the *in vivo* replication of IBV was examined.

## Results

### Human isolate IBV strains are avirulent but spread similarly in wild-type (WT) and TMPRSS2 KO mice

**Figure 1:**
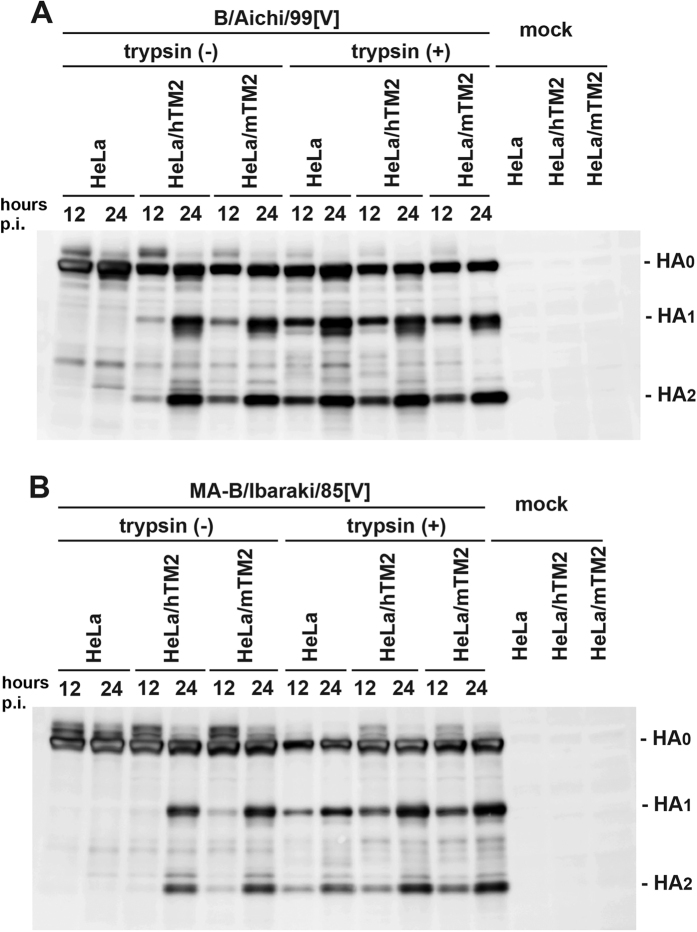
Cleavage of IBV HA by mTMPRSS2 and hTMPRSS2. HeLa cells constitutively expressing mTMPRSS2 or hTMPRSS2 (HeLa/mTM2 and HeLa/hTM2, respectively) and the parental HeLa cells were infected with B/Aichi/99[V] (**A**) or MA-B/Ibaraki/85[V] (**B**) at an MOI of 1.0 in the presence or absence of trypsin. Mock-infected cells were also prepared. At 12 and 24 hours p.i., IBV HA in cells was detected by SDS-PAGE and immunoblotting.

Human TMPRSS2 (hTMPRSS2) has been shown to cleave IBV HA^11^. First, the cleavage activity of mouse TMPRSS2 (mTMPRSS2) for IBV HA was analysed and compared with that of hTMPRSS2. HeLa cells constitutively expressing mTMPRSS2 or hTMPRSS2 (HeLa/mTM2 and HeLa/hTM2, respectively) and the parental HeLa cells were infected with the B/Aichi/99[V] strain (Fig. 1A) or mouse-adapted (MA)-B/Ibaraki/85[V] strain (Fig. 1B). At 12 and 24 hours post-infection (p.i.), polypeptides for IBV HA were detected by SDS-PAGE and immunoblotting. Cleaved forms of HA (HA_1_ and HA_2_) were clearly detected in HeLa/mTM2 and HeLa/hTM2 cells, but not in the parental HeLa cells, infected with either B/Aichi/99[V] or MA-B/Ibaraki/85[V] (Fig. 1A,B). The cleavage efficiency was similar between HeLa/mTM2 and HeLa/hTM2 cells. When the culture media were supplemented with trypsin, cleaved forms of HA were detected in all three cell lines (Fig. 1A,B). These data indicated that mTMPRSS2 cleaves IBV HA as well as hTMPRSS2.

**Figure 2:**
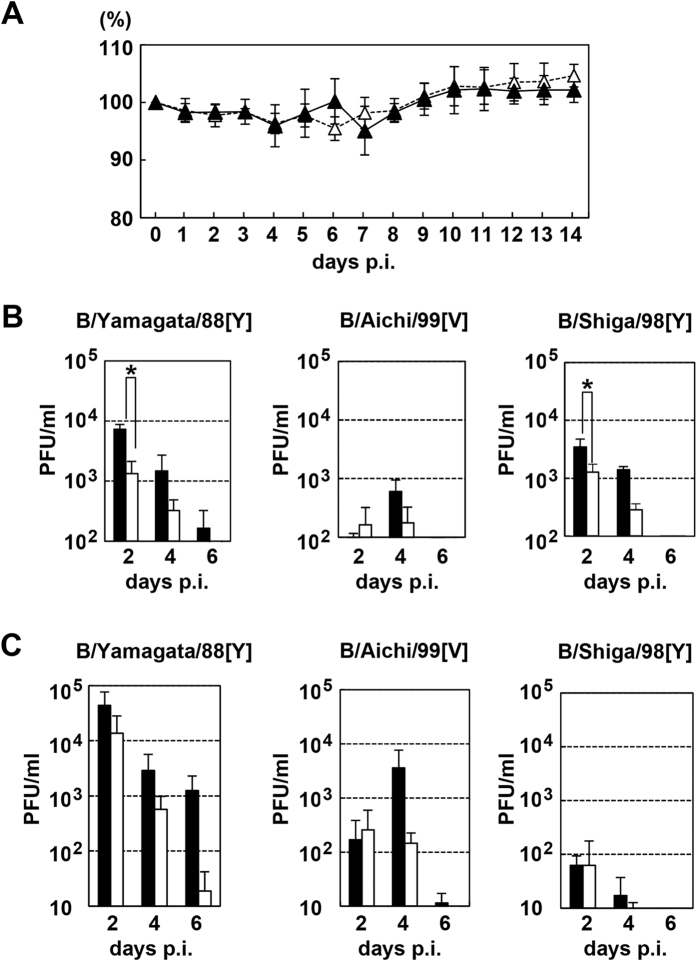
Body weights of WT and TMPRSS2 KO mice infected with human isolates of IBV and virus titres in these mice. (**A**) WT and TMPRSS2 KO mice were intranasally inoculated with 1.6 × 10^4^ PFU of B/Yamagata/88[Y] (*n* = 6). The body weights were measured daily. Filled and open triangles indicate data of WT and TMPRSS2 KO mice, respectively. Data represent means ± SD. (**B**,**C**) WT and TMPRSS2 KO mice (*n* = 5) were intranasally inoculated with 4.6 × 10^3^, 1.6 × 10^4^, and 1.3 × 10^4^ of B/Aichi/99[V], B/Yamagata/88[Y], and B/Shiga/98[Y], respectively. At 2, 4, and 6 days p.i., the virus titres in lung homogenates (**B**) and lung lavage fluids (**C**) were determined. Filled and open bars indicate data of WT and TMPRSS2 KO mice, respectively. Data represent means ± SD. *P < 0.05, significant difference based on a *t*-test.

**Figure 3:**
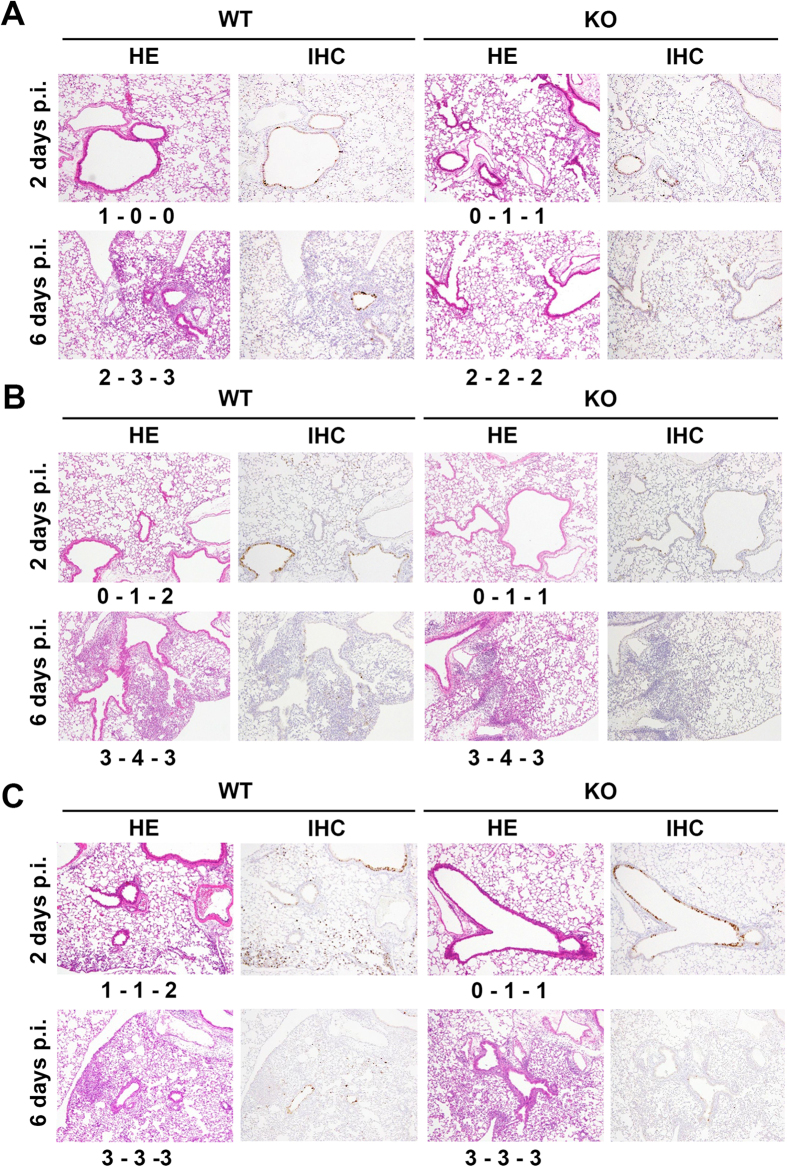
Histopathological findings in the lungs of WT and TMPRSS2 KO mice infected with human isolates of IBV, B/Aichi20/99[V] (**A**), B/Yamagata/88[Y] (**B**), and B/Shiga/98[Y] (**C**). Data obtained by hematoxylin-eosin (HE) staining and immunohistochemistry (IHC) for IBV antigens are shown. Original magnification: ×10. The inflammation scores of individual mice (*n* = 3) are shown: 0, no apparent changes; 1, minimal changes or bronchiolitis; 2, bronchiolitis and/or slight alveolitis; 3, mild alveolitis with neutrophils, monocytes/macrophages, or lymphocytes; 4, moderate alveolitis.

When challenged with B/Aichi/99[V] and B/Shiga/98[Y], both WT and TMPRSS2 KO mice showed little, if any, body weight loss (data not shown). When challenged with B/Yamagata/88[Y] strain, both WT and TMPRSS2 KO mice showed very mild body weight loss (Fig. 2A). Neither WT nor TMPRSS2 KO mice showed any clinical signs (data not shown). The viral titres in lung lavage fluids and lung homogenates of the mice were analysed. Since these IBV strains have not been adapted to grow in mice, the virus titres were generally low (Fig. 2B,C). Nonetheless, the virus titres in TMPRSS2 KO mice were generally slightly lower than those in WT mice (Fig. 2B,C). However, most of the differences between WT and TMPRSS2 KO mice were not significant (*P* > 0.05), with only the data for the lung homogenates of B/Yamagata/88[Y] and B/Shiga/98[Y] at 2 days p.i. showing significant differences (*P* < 0.05) (Fig. 2B). Histopathological analyses demonstrated that the viruses spread moderately in the lungs of both WT and KO mice. Each strain caused slight bronchiolitis at 2 days p.i. and mild alveolitis at 6 days p.i. in both WT and KO mice (Fig. 3). The immunostaining patterns for IBV proteins were similar between WT and KO mice at 2 and 6 days p.i. (Fig. 3). Although these pathological conditions progressed to some extent, they remained mild (Fig. 3). Most importantly, the data revealed no significant differences in the histopathological changes and viral antigen patterns between WT and KO mice infected with IBV.

### The mouse-adapted B/Ibaraki/2/85 strain is fully activated proteolytically *in vivo* and shows full pneumopathogenicity in TMPRSS2 KO mice

**Figure 4:**
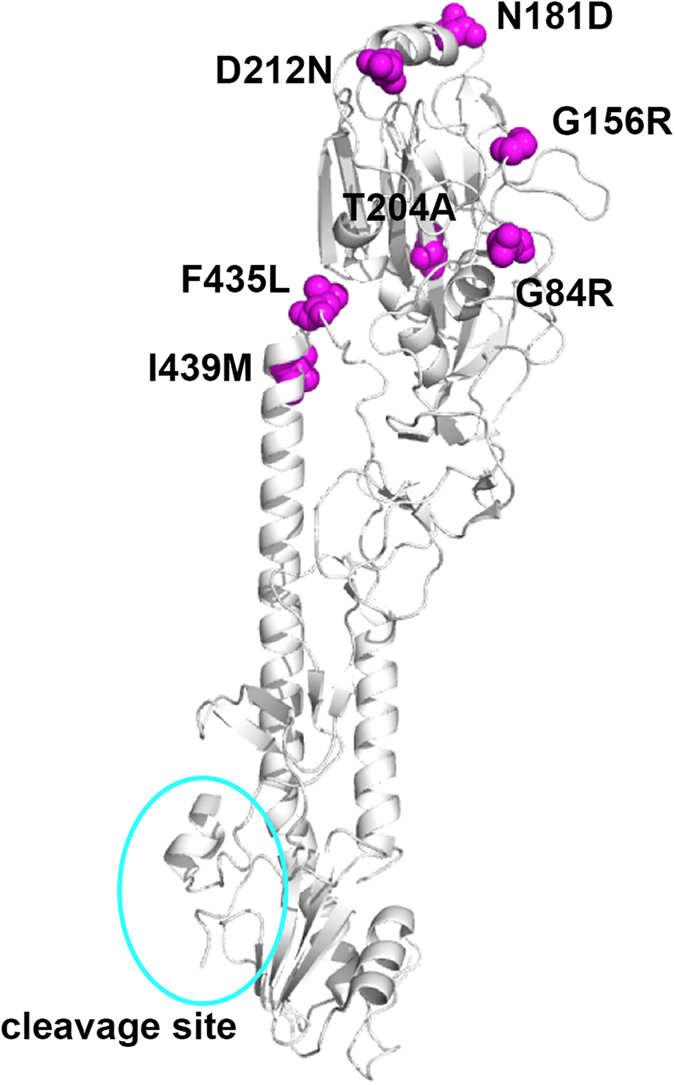
Predicted locations of the mutated amino acid residues in MA-B/Ibaraki/85[V] HA. Based on the structure of B/HongKong/8/73 (PDB 2RFU), the predicted locations of the mutated amino acid residues in MA-B/Ibaraki/85[V] HA are shown. The mutated residues are shown in magenta in a sphere model. The cyan circle indicates the position of the cleavage site.

**Figure 5:**
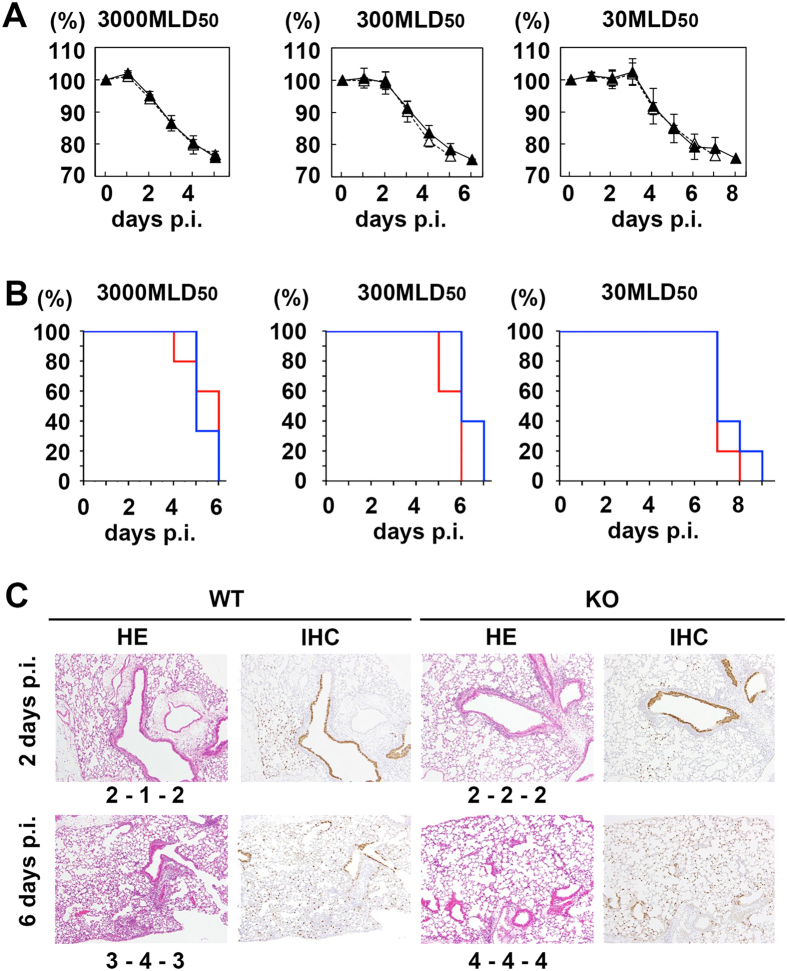
Body weights, survival, and histopathological findings of WT and TMPRSS2 KO mice infected with MA-B/Ibaraki/85[V]. (**A**) WT and TMPRSS2 KO mice were intranasally inoculated with 2.0 × 10^3^, 2.0 × 10^4^, and 2.0 × 10^5^ PFU of MA-B/Ibaraki/85[V] (*n* = 5 or 6). The body weights were measured daily. Filled and open triangles indicate data of WT and TMPRSS2 KO mice, respectively. Data represent means ± SD. (**B**) Survival curves of MA-B/Ibaraki/85[V]-infected mice. Blue and red lines indicate data of WT and TMPRSS2 KO mice, respectively. (**C**) Histopathological findings in the lungs of WT and TMPRSS2 KO mice infected with MA-B/Ibaraki/85[V]. Data obtained by HE staining and IHC for IBV antigens are shown. Original magnification: ×10. The inflammation scores of individual mice (*n* = 3) are shown.

**Figure 6:**
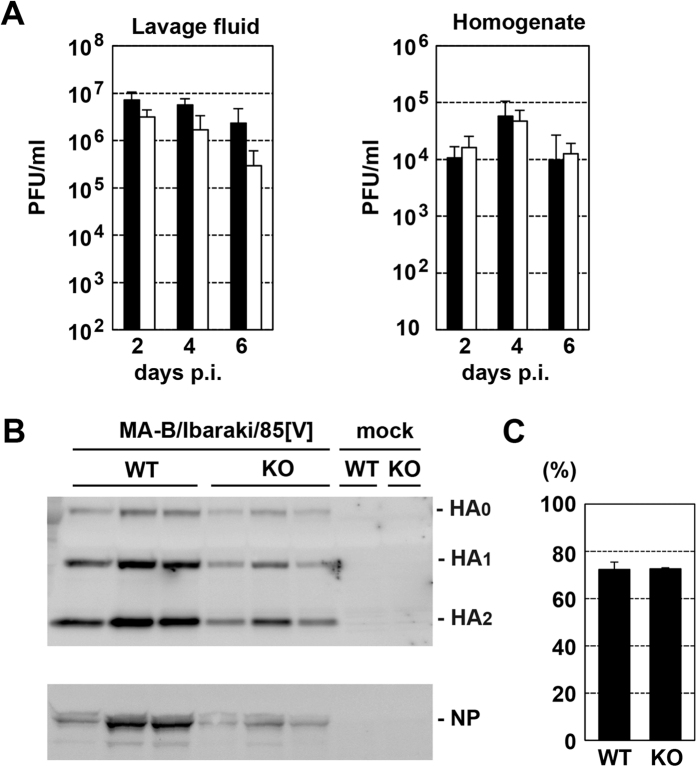
Virus titres and HA cleavage in WT and TMPRSS2 KO mice infected with MA-B/Ibaraki/85[V]. (**A**) WT and TMPRSS2 KO mice (*n* = 3) were intranasally inoculated with 6.7 × 10^3^ PFU of MA-B/Ibaraki/85[V]. Lung lavage fluids and lung homogenates at 2, 4, and 6 days p.i. were subjected to virus titration. Filled and open bars indicate data of WT and TMPRSS2 KO mice, respectively. Data represent means ± SD. (**B**) The HA and NP protein levels in lung lavage fluids at 2 dpi were analysed by SDS-PAGE and immunoblotting. Each lane corresponds to data from an individual mouse. The same amounts of lung lavage fluids were loaded. (**C**) Quantification of cleavage by measuring the chemiluminescent signals for the cleaved HA subunit (HA_1_ and HA_2_) signals to total HA (HA_1_, HA_2_, and HA_0_) signals.

Next, the replication capacity and virulence of the MA-B/Ibaraki/85[V] strain were analysed. Since the adaptation to mice must cause mutations in the viral genome, the genomic sequences of the parental B/Ibaraki/85[V] and MA-B/Ibaraki/85[V] strains were determined. The genomic sequence of the parental B/Ibaraki/85[V] strain was deposited in the GISAID database with the following GISAID Isolate ID numbers: EPI_ISL_219717; PB2: EPI749072; PB1: EPI749073; PA: EPI749074; HA: EPI749075; NP: EPI749076; NA: EPI749077; MP: EPI749078; NS: EPI749079. The mutations acquired by MA-B/Ibaraki/85[V] are summarized in Table 1. Seven amino acid changes were found in the HA protein. The residues were mapped on the IBV HA structure (PDB 2RFU). All of these residues were located in the globular head domain at positions far distant from the cleavage site (Fig. 4). Both WT and KO mice showed severe body weight loss and required euthanasia, when challenged with MA-B/Ibaraki/85[V]. Similar kinetics of body weight loss were observed for WT and KO mice (Fig. 5A). The survival rates were also similar between WT and TMPRSS2 KO mice (Fig. 5B). At 2 days p.i., the lungs of both WT and KO mice showed similar levels of bronchiolitis with slight alveolitis, and viral antigens were detected throughout the bronchial epithelia (Fig. 5C). At 6 days p.i., moderate alveolitis had progressed in the lungs of both WT and KO mice with viral antigens spreading throughout the lungs (Fig. 5C). The virus titres in the lung lavage fluids and lung homogenates from both WT and KO mice were high during the observation period, although the titres in the lung lavage fluids in WT mice tended to be several-fold higher than those in KO mice (Fig. 6A). However, the differences were not significant (*P* < 0.05). The extent of activation *in vivo* of the progeny viruses was analysed. Even in KO mice, the infectivity titres were not restored significantly by *in vitro* trypsin treatment (Table 2), indicating that the majority of MA-B/Ibaraki/85[V] was produced in an almost fully-activated form in the lungs of both WT and KO mice. To obtain evidence of HA cleavage *in vivo*, lung lavage fluids collected from infected WT and KO mice (*n* = 3 for each) were analysed by SDS-PAGE and immunoblotting. Generally, the HA signal intensities were greater in WT mice than in KO mice (Fig. 6B), consistent with the greater amounts of progeny viruses in WT mice compared with KO mice (Fig. 6A). However, it was clearly shown that HA was already cleaved into subunits (HA_1_ and HA_2_) in both WT and KO mice (Fig. 6B). The NP signals were also greater in WT mice than in TMPRSS2 KO mice (Fig. 6B). The extents of cleavage estimated by the signal intensities of HA_0_ and the cleaved HA subunits (HA_1_ and HA_2_) were similar in WT and TMPRSS2 KO mice (Fig. 6C). Taken together, in both WT and KO mice, MA-B/Ibaraki/85[V], whose HA possesses a monobasic cleavage site, was almost fully activated proteolytically and replicated in multiple steps in the lungs, resulting in pneumonia and exhibiting high pathogenicity.

## Discussion

Our data show a clear difference in the *in vivo* protease specificity between IAV and IBV. Many studies have been performed to identify the proteases responsible for HA activation, and many candidate proteases have been reported^12^. Several TTSPs, such as TMPRSS2^13^,^14^, HAT^13^, TMPRSS4^15^,^16^, matriptase^17^,^18^, MSPL^19^, and DESC1^19^, were also shown to activate IAV strains, although certain TTSPs may activate specific HA subtypes. Recent studies have provided concrete evidence that among these proteases, TMPRSS2 is the main protease for monobasic HA cleavage in IAV strains *in vivo*^8^,^9^,^10^. Meanwhile, high-pathogenic (HP) avian IAV strains possessing a multibasic HA cleavage site use furin and pro-protein convertase 5/6^20^,^21^. As these proteases are ubiquitously expressed in the *trans*-Golgi network, HP strains have the potential to spread in a variety of organs and tissues, causing lethal infections in domestic poultry. Thus, HP IAV strains show high virulence, even toward TMPRSS2 KO mice^8^,^10^. Compared with efforts for IAV, only limited data are available for HA cleavage in IBV strains. Previous studies have demonstrated different levels of trypsin-dependency between IAV and IBV for growth in specific MDCK cell lines^22^,^23^. Noma *et al*.^22^ suggested that IBV HA has higher sensitivity to an endoprotease(s), currently unidentified, than IAV HA, or that specific MDCK cell lines express an endoprotease that only activates IBV HA. Further analyses using cell cultures demonstrated that TMPRSS2 activates IBV HA as well as IAV HA^11^. Therefore, IBV strains may utilize TMPRSS2 for HA cleavage *in vivo*, similar to IAV strains. Nevertheless, our present data clearly demonstrate that TMPRSS2 is dispensable for HA cleavage and pathogenicity of IBV in mice. However, the virus titres in TMPRSS2 KO mice were slightly lower than those in WT mice. Therefore, potential anti-IAV drugs targeting TMPRSS2 are less effective for IBV. Under certain experimental conditions, H3N2 also showed the ability to use a TMPRSS2-independent HA activation mechanism through loss of an oligosaccharide at the HA stalk region^24^. The loss of this oligosaccharide likely improved the accessibility of an alternative host protease to the loop containing the HA cleavage site of IAV. The HA protein of IBV also possesses oligosaccharides at the stalk region and near the cleavage site^25^, but our data in the present study show that they do not limit the activation protease to TMPRSS2. The stalk oligosaccharide of IBV HA attached *in vivo* may be incompatible with the HA cleavage by TMPRSS2. Because the protease specificity of viruses determines virus tropism and pathogenicity^26^,^27^, identification of the protease(s) responsible for IBV HA cleavage may reveal a molecular basis for the different biological features between IAV and IBV, and contribute to the development of anti-IBV drugs.

## Materials and Methods

### Ethics statement

All experiments with animals were performed in strict accordance with the Animal Experimentation Guidelines of the National Institute of Infectious Diseases, and the protocol was approved by the Institutional Animal Care and Use Committee of the institute.

### Mice

TMPRSS2 KO mice were reported previously^10^. Briefly, the mice were generated from *TMPRSS2* gene KO C57BL/6 ES cells (KOMP Repository Knockout Mouse Project; Product ID: VG13341) and have a complete C57BL/6 genetic background. WT C57BL/6 mice were purchased from Japan SLC.

### Viruses and cells

Four IBV human isolates, two from the Victoria lineage, B/Aichi20/99 (B/Aichi/99[V]) and B/Ibaraki/2/85 (B/Ibaraki/85[V]), and two from the Yamagata lineage, B/Yamagata/16/88 (B/Yamagata/88[Y]) and B/Shiga/T30/98 (B/Shiga/98[Y]), and a mouse-adapted (MA)-B/Ibaraki/85[V], were used. The human strains were isolated in MDCK cells or embryonated eggs. The adaptation process of the MA-B/Ibaraki/85[V] strain was reported previously^28^. HeLa cells constitutively expressing mouse TMPRSS2 and human TMPRSS2 (HeLa/mTM2 and HeLa/hTM2) were reported previously^10^.

### Infection of mice with human isolates of IBV

WT and TMPRSS2 KO mice (6–7-week-old) were infected intranasally with 4.6 × 10^3^, 1.6 × 10^4^, and 1.3 × 10^4^ PFU of B/Aichi/99[V], B/Yamagata/88[Y], and B/Shiga/98[Y], respectively. For mock infection, the same volume (20 μL) of PBS was used. The body weight and clinical signs were monitored daily. For histopathological analysis, WT and TMPRSS2 KO mice were euthanized and autopsied at 2 days p.i. (*n* = 3) or 6 days p.i. (*n* = 3). The viral antigens of IBV were detected using a mouse antiserum against IBV NP (Lot. N179). This antiserum was obtained from BALB/c mice immunized intraperitoneally with inactivated IBV virions (Victoria lineage) using the Sigma adjuvant system (Sigma-Aldrich). The inflammation levels in individual mice were scored as follows: 0, no apparent changes; 1, minimal changes or bronchiolitis; 2, bronchiolitis and/or slight alveolitis; 3, mild alveolitis with neutrophils, monocytes/macrophages, or lymphocytes; 4, moderate alveolitis. The cut-off in body weight loss for euthanasia was 25%. Lung lavage fluids and lung homogenates were collected at 2, 4, and 6 days p.i. and subjected to a standard plaque assay.

### Infection of mice with MA-B/Ibaraki/85[V]

WT and TMPRSS2 KO mice (6–7-week-old) were infected intranasally with 2.0 × 10^3^, 2.0 × 10^4^, and 2.0 × 10^5^ PFU of MA-B/Ibaraki/85[V]. These doses of MA-B/Ibaraki/85[V] correspond to 30, 300, and 3,000 mouse lethal dose 50 (MLD_50_) for WT mice. The body weight and clinical signs were monitored daily. For histopathological analysis, similar mice were also infected intranasally with 6.7 × 10^3^ PFU of MA-B/Ibaraki/85[V] and autopsied at 2 days p.i. (*n* = 3) or 6 days p.i. (*n* = 3).

### Analysis of infectious virus titres activated *in vivo*

WT and TMPRSS2 KO mice (*n* = 3) were infected with 2.0 × 10^3^ PFU of MA-B/Ibaraki/85[V] and lung lavage fluids were collected at 2, 4, and 6 days p.i. Monolayers of MDCK cells were infected with serially diluted lung lavage fluid and lung homogenate samples for 1 hour at 4 °C, washed twice with PBS, overlaid with MEM/1% agarose, and incubated for 24 hours at 37 °C to allow the viruses to enter the cells. Trypsin was omitted to avoid HA cleavage before virus entry. At 24 hours p.i., the cell monolayers were additionally overlaid with MEM/1% agarose supplemented with 4.0 μg/mL of trypsin to allow plaque formation. To determine the infectious titres including viruses that had not been activated *in vivo* but possessed an infectious potential, virus samples were treated with 2.0 μg/mL of trypsin, and subjected to a standard plaque assay.

### Immunoblotting

WT and TMPRSS2 KO mice were infected with 6.7 × 10^3^ PFU of MA-B/Ibaraki/85[V] (*n* = 3) or mock-infected (*n* = 1). Lung lavage fluids were collected at 2 days p.i. HeLa/mTMPRSS2, HeLa/hTMPRSS2, and parental HeLa cells were infected with B/Yamagata/88[Y], B/Shiga/98[Y], MA-B/Ibaraki/85[V], and parental B/Ibaraki/85[V] at a multiplicity of infection (MOI) of 1.0, and collected at 12 and 24 hours p.i. The lung lavage fluids and cells were lysed with lysis buffer to make a final solution containing 150 mM NaCl, 50 mM Tris-HCl (pH 7.5), 4 mM EDTA, 0.1% sodium deoxycholate, 1% Nonidet P-40, and 0.1% SDS. The polypeptides were separated by SDS-PAGE in 10–20% polyacrylamide gels (e-PAGEL, E-T/R1020L; ATTO), and blotted onto PVDF membranes (WSE-4051; ATTO). Rabbit antisera raised against IBV HA (#11053-RP01; Sino Biological Inc.) and NP (#GTX128539; GeneTex Inc.) were used for detection of HA and NP, respectively.

## Additional Information

**How to cite this article**: Sakai, K. *et al*. TMPRSS2 Independency for Haemagglutinin Cleavage *In Vivo* Differentiates Influenza B Virus from Influenza A Virus. *Sci. Rep.* **6**, 29430; doi: 10.1038/srep29430 (2016).

## Figures and Tables

**Table 1:** Nucleotide and amino acid differences between the parental (PT) B/Ibaraki/95[V] and MA-B/Ibaraki/85[V] strains.

Gene/segment	Nucleotide			Protein	Amino acid		
	Position^a^	PT	MA		Position	PT	MA
PB2	226	A	G	PB2	76	R	G
PB1		None					
PA	779	G	A	PA	260	G	E
	1160	A	G		387	Y	C
HA	250	G	A	HA	84	G	R
	466	G	G, A		156	G	G, R
	480	T	C		160	synonymous	
	541	A	A, G		181	N	N, D
	610	A	A, G		204	T	T, A
	634	G	G, A		212	D	D, N
	1303	T	C		435	F	L
	1317	A	A, G		439	I	I, M
NP	1176	A	T	NP	392	synonymous	
	1251	A	G		417	synonymous	
	1543	G	A		515	G	N
	1544	G	A		515	G	N
NA	1200	T	C	NA	400	synonymous	
	1371	C	T		457	synonymous	
M	847	G, A	G	BM2	34	G, E	G
NS	112	A	G	NS1	38	R	G

^a^The number of the first nucleotide of the initiation codons for PB1, PB2, PA, HA, NP, NA, M1, and NS1 proteins is set to ‘nucleotide position 1′. When two nucleotides are detected at one position, both nucleotides and two predicted amino acids are shown.

**Table 2:** The extent of activation *in vivo* of the progeny viruses in lung lavage fluids of mice infected with MA-B/Ibaraki/85[V].

	Titre (PFU/ml)				
	Mouse	*n* = *3*	Trypsin+	Trypsin−	Activation (%)^a^
2 days p.i.	WT	Means	7.2 × 10^6^	6.8 × 10^6^	94.4
		SD	3.3 × 10^6^	1.9 × 10^6^	
	KO	Means	3.2 × 10^6^	4.7 × 10^6^	146.9
		SD	1.3 × 10^6^	0.3 × 10^6^	
4 days p.i.	WT	Means	5.7 × 10^6^	1.3 × 10^6^	22.8
		SD	1.9 × 10^6^	0.3 × 10^6^	
	KO	Means	1.7 × 10^6^	7.7 × 10^5^	45.3
		SD	1.6 × 10^6^	6.5 × 10^5^	
6 days p.i.	WT	Means	2.4 × 10^6^	7.2 × 10^5^	30.0
		SD	2.3 × 10^6^	2.8 × 10^5^	
	KO	Means	2.9 × 10^5^	1.9 × 10^5^	65.5
		SD	3.1 × 10^5^	1.5 × 10^5^	

^a^The percentage of virus titres activated *in vivo* (Trypsin−) in total virus titres activated *ex vivo* (Trypsin+).

## References

[b1] Wright P. F., Neumann G. & Kawaoka Y. in Fields Virology (eds Knipe D. M. . ) 1186–1243 (Lippincott Williams & Wilkins, 2013).

[b2] Tong S. . New world bats harbor diverse influenza A viruses. Plos Pathog 9, e1003657, 10.1371/journal.ppat.1003657 (2013). 24130481 PMC3794996

[b3] Tong S. . A distinct lineage of influenza A virus from bats. Proc Natl Acad Sci USA 109, 4269–4274, 10.1073/pnas.1116200109 (2012). 22371588 PMC3306675

[b4] Kanegae Y. . Evolutionary pattern of the hemagglutinin gene of influenza B viruses isolated in Japan: cocirculating lineages in the same epidemic season. J Virol 64, 2860–2865 (1990). 2335820 10.1128/jvi.64.6.2860-2865.1990 PMC249468

[b5] Rota P. A. . Cocirculation of two distinct evolutionary lineages of influenza type B virus since 1983. Virology 175, 59–68 (1990). 2309452 10.1016/0042-6822(90)90186-u

[b6] Osterhaus A. D. . Canine distemper virus in seals. Nature 335, 403–404, 10.1038/335403a0 (1988). 3419515

[b7] Ran Z. . Domestic pigs are susceptible to infection with influenza B viruses. J Virol 89, 4818–4826, 10.1128/JVI.00059-15 (2015). 25673727 PMC4403465

[b8] Hatesuer B. . Tmprss2 is essential for influenza H1N1 virus pathogenesis in mice. Plos Pathog 9, e1003774, 10.1371/journal.ppat.1003774 (2013). 24348248 PMC3857797

[b9] Tarnow C. . TMPRSS2 is a host factor that is essential for pneumotropism and pathogenicity of H7N9 influenza A virus in mice. J Virol 88, 4744–4751, 10.1128/JVI.03799-13 (2014). 24522916 PMC3993819

[b10] Sakai K. . The host protease TMPRSS2 plays a major role in *in vivo* replication of emerging H7N9 and seasonal influenza viruses. J Virol 88, 5608–5616, 10.1128/JVI.03677-13 (2014). 24600012 PMC4019123

[b11] Bottcher-Friebertshauser E. . Hemagglutinin activating host cell proteases provide promising drug targets for the treatment of influenza A and B virus infections. Vaccine 30, 7374–7380, 10.1016/j.vaccine.2012.10.001 (2012). 23072892

[b12] Kido H. . Host envelope glycoprotein processing proteases are indispensable for entry into human cells by seasonal and highly pathogenic avian influenza viruses. Journal of molecular and genetic medicine: an international journal of biomedical research 3, 167–175 (2008). 19565019 PMC2702071

[b13] Bottcher E. . Proteolytic activation of influenza viruses by serine proteases TMPRSS2 and HAT from human airway epithelium. J Virol 80, 9896–9898 (2006). 16973594 10.1128/JVI.01118-06 PMC1617224

[b14] Garten W., Matrosovich M., Matrosovich T., Eickmann M. & Vahhabzadeh A. Cleavage of influenza virus hemagglutinin by host cell protease. Int Congr Ser 1263, 218–221 (2004).

[b15] Chaipan C. . Proteolytic activation of the 1918 influenza virus hemagglutinin. J Virol 83, 3200–3211, 10.1128/JVI.02205-08 (2009). 19158246 PMC2655587

[b16] Kuhn N. . The Proteolytic Activation of (H3N2) Influenza A Virus Hemagglutinin Is Facilitated by Different Type II Transmembrane Serine Proteases. J Virol 90, 4298–4307, 10.1128/JVI.02693-15 (2016). 26889029 PMC4836353

[b17] Beaulieu A. . Matriptase Proteolytically Activates Influenza Virus and Promotes Multicycle Replication in the Human Airway Epithelium. J Virol, 10.1128/JVI.03005-12 (2013). PMC3624356 23365447

[b18] Hamilton B. S., Gludish D. W. & Whittaker G. R. Cleavage activation of the human-adapted influenza virus subtypes by matriptase reveals both subtype and strain specificities. J Virol 86, 10579–10586, 10.1128/JVI.00306-12 (2012). 22811538 PMC3457293

[b19] Zmora P. . DESC1 and MSPL activate influenza A viruses and emerging coronaviruses for host cell entry. J Virol 88, 12087–12097, 10.1128/JVI.01427-14 (2014). 25122802 PMC4178745

[b20] Stieneke-Grober A. . Influenza virus hemagglutinin with multibasic cleavage site is activated by furin, a subtilisin-like endoprotease. EMBO J 11, 2407–2414 (1992). 1628614 10.1002/j.1460-2075.1992.tb05305.x PMC556715

[b21] Horimoto T., Nakayama K., Smeekens S. P. & Kawaoka Y. Proprotein-processing endoproteases PC6 and furin both activate hemagglutinin of virulent avian influenza viruses. J Virol 68, 6074–6078 (1994). 8057485 10.1128/jvi.68.9.6074-6078.1994 PMC237016

[b22] Noma K. . Endogenous protease-dependent replication of human influenza viruses in two MDCK cell lines. Arch Virol 143, 1893–1909 (1998). 9856079 10.1007/s007050050428

[b23] Lugovtsev V. Y., Melnyk D. & Weir J. P. Heterogeneity of the MDCK cell line and its applicability for influenza virus research. Plos One 8, e75014, 10.1371/journal.pone.0075014 (2013). 24058646 PMC3772841

[b24] Sakai K. . A mutant H3N2 influenza virus uses an alternative activation mechanism in TMPRSS2 knockout mice by loss of an oligosaccharide in the hemagglutinin stalk region. J Virol 89, 5154–5158, 10.1128/JVI.00124-15 (2015). 25673722 PMC4403495

[b25] Wang Q., Cheng F., Lu M., Tian X. & Ma J. Crystal structure of unliganded influenza B virus hemagglutinin. J Virol 82, 3011–3020, 10.1128/JVI.02477-07 (2008). 18184701 PMC2259021

[b26] Nagai Y. Virus activation by host proteinases. A pivotal role in the spread of infection, tissue tropism and pathogenicity. Microbiol Immunol 39, 1–9 (1995). 7783672 10.1111/j.1348-0421.1995.tb02161.x

[b27] Rott R., Klenk H. D., Nagai Y. & Tashiro M. Influenza viruses, cell enzymes, and pathogenicity. Am J Respir Crit Care Med 152, S16–19 (1995). 7551406 10.1164/ajrccm/152.4_Pt_2.S16

[b28] Kikuta K. . Cross-protection against influenza B type virus infection by intranasal inoculation of the HA vaccines combined with cholera toxin B subunit. Vaccine 8, 595–599 (1990). 1965078 10.1016/0264-410x(90)90016-f

